# X-Ray Diffraction Tomography Recovery of the 3D Displacement-Field Function of the Coulomb-Type Point Defect in a Crystal

**DOI:** 10.1038/s41598-019-50833-6

**Published:** 2019-10-02

**Authors:** F. N. Chukhovskii, P. V. Konarev, V. V. Volkov

**Affiliations:** 1 Shubnikov Institute of Crystallography, Federal Scientific Research Centre “Crystallography and Photonics”, Russian Academy of Sciences, Moscow, 119333 Russia; 2 National Research Centre “Kurchatov Institute”, Moscow, 123182 Russia

**Keywords:** Structure of solids and liquids, Surfaces, interfaces and thin films

## Abstract

A successive approach to the solution of the inverse problem of the X-ray diffraction tomography (XRDT) is proposed. It is based on the *semi-kinematical* solution of the *dynamical* Takagi–Taupin equations for the σ-polarized diffracted wave amplitude. Theoretically, the case of the Coulomb-type point defect in a single crystal Si(111) under the exact conditions of the symmetric Laue diffraction for a set of the tilted X-ray topography 2D-images (2D projections) is considered provided that the plane-parallel sample is rotated around the diffraction vector [$\bar{{\bf{2}}}$20]. The iterative simulated annealing (SA) and quasi-Newton gradient descent (qNGD) algorithm codes are used for a recovery of the 3D displacement-field function of the Coulomb-type point defect. The computer recovery data of the 3D displacement-field function related to the one XRDT 2D projection are presented. It is proved that the *semi-kinematical* approach to the solution of the *dynamical* Takagi–Taupin equations is effective for recovering the 3D displacement-field function even for the one XRDT 2D projection.

## Introduction

As is well-known^1–5^, the X-ray diffraction topography method is a highly sensitive nondestructive diagnostics method for observing various crystal lattice defects like striations, grain boundaries, stacking faults, single clusters, and dislocations. All these defects change the positions of individual atoms of crystal cell relative to their regular position. The real crystal structure can be studied by the Laue diffraction topography method that provides the 2D image (2D projection) of crystal with defects. Based on the analytical approximate solutions of the X-ray optics problem by the stationary method (*cf*. the geometrical X-ray optics method in^6^), the theoretical fundamentals for an analysis of the X-ray diffraction scattering from macroscopical crystalline objects are elaborated and widely used (see^3,6,7^, and references therein for details). In particular, in^7^, in the case of the X-ray diffraction from the crystals with defects, the basic principles and theoretical analysis of the X-ray coherent diffraction intensity and incoherent (diffuse) scattering intensity, the latter is caused by the X-ray scattering from the statistical ensemble of crystal lattice defects, are comprehensively presented. In practice, to interpret and analyze the experimental 2D images of crystals with lattice defects, the defect images are compared with the corresponding ones that can be evaluated using numerical methods for solving the *dynamical* Takagi–Taupin equations^1–3,8–10^.

**Figure 1:**
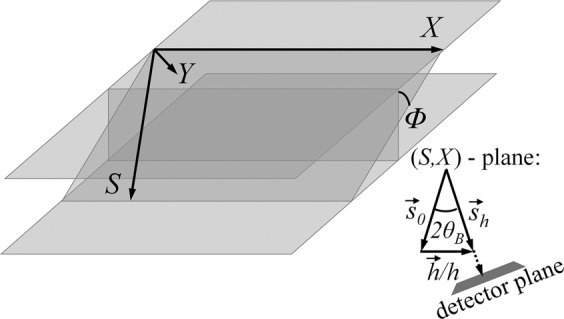
Original coordinate system (*X, S*) for the inclined X-ray diffraction geometry; 0*Y*-axis is perpendicular to the (*X, S*)-plane. *Φ* is the crystal rotation angle around the diffraction vector ***h*** parallel to the 0*X*-axis. The detector plane is perpendicular to the unit vector ***s***_h_ = **k**_h|_/k along the diffracted wave propagation.

In the last 20 years, the X-ray diffraction tomography (XRDT) method has been widely applied to investigate the real crystalline materials. In this method, the plate-parallel crystal sample is rotated around an axis perpendicular to a set of reflection crystal planes, namely: the rotation axis is chosen to coincide with the diffraction vector ***h***. Correspondingly, a set of the XRDT projections can be obtained at different rotation angle values. Each of these XRDT 2D projections corresponds to a certain orientation of the X-ray diffraction planes relative to the intrinsic Cartesian system of coordinates of the crystal sample as it is shown in Fig. 1.

The first XRDT experiments have been successfully performed on the synchrotron radiation sources ESRF^11^ and SPring-8^12^. Then, similar experiments were carried out on the laboratory setups using the X-ray characteristic line radiation^13,14^. Using a complete set of experimental XRDT 2D projections, the corresponding computer recovery of the 3D dislocation images has been carried out based on the modified conventional iterative tomography algorithm codes (*cf*.^13–15^).

It is obvious, that the 3D computer image recovery based on any experimental XRDT data is connected equally, if not to a greater extent, with the same difficulties as the interpretation of the XRDT 2D projections on the X-ray diffraction topograms. They are due to the complicated contrast mechanisms of the image formation related to various regions around defects in crystals^1–3^. In this respect, it is important to find out approximate analytical solutions of the *dynamical* Takagi–Taupin equations, which would allow describing some features of the X-ray diffraction topograms related to particular regions in the neighborhood of single defects in crystals. As is expected, this is a key factor for solving the inverse XRDT problem, in particular, for the 3D recovery of the displacement-field function near around single defects.

In the present study, an approximate analytical solution of the *dynamical* Takagi–Taupin equations is found out that seems to represent by itself a principal point to the inverse XRDT problem. Such the *semi-kinematical* approximation for the diffracted wave *E*_**h**_(**r**) is used for recovering the 3D displacement-field function $f({\bf{r}}-{{\bf{r}}}_{0})={\boldsymbol{h}}\cdot {\boldsymbol{u}}({\bf{r}}-{{\bf{r}}}_{0})$ in the case of the Coulomb-type point defect (**r**_0_ is the radius-vector of a point defect in a crystal). By applying iterative simulated annealing (SA)^16^ and quasi-Newton gradient descent (qNGD) algorithm codes^17,18^, we will show that in the case of the symmetric Laue diffraction of the X-ray characteristic *MoK*_α1_-radiation with the diffraction vector ***h*** = [$\bar{2}$20] from a single crystal Si(111) the 3D displacement-field point defect function $f({\bf{r}}-{{\bf{r}}}_{0})={\bf{h}}\cdot {\bf{u}}({\bf{r}}-{{\bf{r}}}_{0})$ can be recovered.

Note that the first attempt to recover the 3D displacement-field point defect function was made in^19^ using the so-called simultaneous algebraic reconstruction technique (SART) algorithm (*cf*.^13^). Unfortunately, the SART solutions turn out to converge only for a limited number of voxels (no more than 5 × 5 × 5 = 125 voxels) near around a point defect in a crystal.

## Results and Discussion

### The 2D diffraction imaging of a point defect. *Semi-kinematical* approximation

In this section, one derives the solution of the *dynamical* Takagi–Taupin equations in the *semi-kinematical* approximation, which seems to be effective for recovering the 3D displacement-field function using the inclined XRDT 2D projections data.

As is well-known^1–3^, a direct image of defect in a crystal is due to the interbranch scattering of the X-ray Bloch waves in the strongly distorted region in the immediate vicinity of a defect, which can be interpreted as the *kinematical* scattering of Bloch waves propagating in a perfect crystal far from the defect central region. This assertion was confirmed by numerous numerical calculations (see, *e.g*.^2,3,10^).

For simplicity, in order to avoid cumbersome formulas and calculations, we restrict ourselves to the case of propagation of a σ-polarized X-ray wave-field in a non-absorbing (thin) crystal “on average” oriented in the exact Bragg position, $|{{\boldsymbol{k}}}_{0}+{\boldsymbol{h}}|=|{{\boldsymbol{k}}}_{0}|=k$, where ***k***_0_ is the wave vector of the monochromatic X-ray plane wave incident onto a crystalline sample. In this case, the propagation of the total X-ray wave-field in a distorted crystal under conditions of the symmetric two-wave Laue diffraction is set in the form of the *dynamical* Takagi–Taupin equations (*cf*.^8,9^)

1 $$\begin{array}{rcl}-\frac{2i}{k}\frac{\partial {E}_{0}}{\partial {s}_{0}} & = & {{\rm{\chi }}}_{0}{E}_{0}+{{\rm{\chi }}}_{\bar{h}}{{\rm{e}}}^{if({\bf{r}})}{E}_{h}-\frac{2i}{k}{\rm{\delta }}({s}_{0}+{s}_{h})\\ -\frac{2i}{k}\frac{\partial {E}_{h}}{\partial {s}_{h}} & = & {{\rm{\chi }}}_{0}{E}_{h}+{{\rm{\chi }}}_{h}{{\rm{e}}}^{-if({\bf{r}})}{E}_{0}\end{array},\,f({\bf{r}})={\boldsymbol{h}}\cdot {\boldsymbol{u}}({\boldsymbol{r}}),$$

with the boundary conditions on the entrance crystal surface (${s}_{0}=-\,{s}_{h}$)

2 $$\begin{array}{rcl}{E}_{0}(-{s}_{h},{s}_{h}) & = & 1,\\ {E}_{h}(-{s}_{h},{s}_{h}) & = & 0.\end{array}$$

Hereabove, in Eq. (1), $f({\bf{r}}),\,f({\bf{r}})={\boldsymbol{h}}\cdot {\boldsymbol{u}}({\boldsymbol{r}})$, is the 3D displacement-field function describing the distorted crystal lattice of the crystal with defects and the oblique coordinates *s*_0_, *s*_*h*_ are directed along the wave-vectors of transmitted and diffracted waves, respectively.

Note that the boundary conditions (2) are automatically followed from the modified Eq. (1) with the added term $-\frac{2i}{k}{\rm{\delta }}({s}_{0}+{s}_{h})$ in the right-hand side of the first line equation. Using the well-known substitutions ${E}_{0}\to {E}_{0}\,{{\rm{e}}}^{ik{{\rm{\chi }}}_{0}\frac{{s}_{0}+{s}_{h}}{2}},{E}_{h}\to {E}_{h}\,{{\rm{e}}}^{ik{{\rm{\chi }}}_{0}\frac{{s}_{0}+{s}_{h}}{2}-if({\boldsymbol{r}})}$ and retaining the previous designations for the amplitudes of transmitted and diffracted waves, ${E}_{0}({s}_{0},{s}_{h}),{E}_{h}({s}_{0},{s}_{h})$, one can easily show that the diffracted amplitude ${E}_{h}({S}_{0},{S}_{h})$ satisfies the inhomogeneous differential hyperbolic-type second-order equation in the partial derivatives over the dimensionless variables *S*_0_ and *S*_h_ (*S*_0_ = ***s***_0_/Λ, *S*_h_ = ***s***_h_/Λ)

3 $$\begin{array}{rcl}\frac{{\partial }^{2}{E}_{h}}{\partial {S}_{h}\partial {S}_{0}}+{{\Gamma }}^{2}{E}_{h} & = & i\frac{\partial }{\partial {S}_{0}}[\frac{\partial }{\partial {S}_{h}}[f({\boldsymbol{r}})]{E}_{h}]+i{{\Gamma }}_{h}\delta ({S}_{0}+{S}_{h}),\\  &  & ({\Gamma }^{2}\equiv {{\Gamma }}_{h}\,{{\Gamma }}_{\bar{h}},\,{\rm{Re}}({\Gamma })=1){\rm{.}}\end{array}$$

Hereafter, ${E}_{0,h}({S}_{0},{S}_{h})$ are the amplitudes of transmitted and diffracted waves in the crystalline medium; correspondingly, *S*_0_ and *S*_h_ are the oblique coordinates along the unit vectors of ${{\boldsymbol{s}}}_{0}=\frac{{{\boldsymbol{k}}}_{0}}{k}$, ${{\boldsymbol{s}}}_{h}=\frac{{{\boldsymbol{k}}}_{h}}{k}$. In the Eq. (3) the following notations for dimensionless variables *S*_0_ and *S*_h_, complex parameters Γ_*h*_, ${\Gamma }_{\overline{h}}$ and $\Lambda$ are introduced

4 $${S}_{0}=\frac{{s}_{0}}{{\Lambda }},{S}_{h}=\frac{{s}_{h}}{{\Lambda }},{{\Gamma }}_{h}=\frac{\pi {\Lambda }}{\lambda }C{\chi }_{h},{\Gamma }_{\bar{h}}=\frac{\pi {\Lambda }}{\lambda }C{\chi }_{\bar{h}},\Lambda =\frac{\lambda }{\pi {[{Re}({C}^{2}{\chi }_{h}{\chi }_{\bar{h}})]}^{1/2}},$$

where *C* is the X-ray polarization factor equal to 1 for σ-polarization and *cos* 2*θ*_*B*_ for π-polarization.

Further, we will choose a plane-parallel single crystal Si(111) sample with diffraction vector ***h*** = [$\bar{2}$20]; the wavelength λ of incident characteristic X-ray radiation equal to 0.071 nm of the characteristic X-ray *Mo K*_α1_-radiation, photon energy of 17.48 keV. Correspondingly, the susceptibility coefficients *Re*(χ_*h*_) = *Re*(χ_−*h*_) = −1.921 × 10^−6^ and *Im*(χ_*h*_) = *Im*(χ_-*h*_) = 1.55 × 10^−8^; the Bragg angle *θ*_B_ = 10,65°; the X-ray extinction length $\ell \,=\pi {\Lambda }cos{\theta }_{B}$, *ℓ* = 36.287 μm; *C* = 1 for the σ-polarized X-ray plane wave. The vector elastic displacement field of a Coulomb-type point defect, ${\bf{u}}({\bf{r}}-{{\bf{r}}}_{0})$ has a form

5 $${\bf{u}}({\bf{r}}-{{\bf{r}}}_{0})=\frac{F}{4{\rm{\pi }}}\frac{{\bf{r}}-{{\bf{r}}}_{0}}{{|{\bf{r}}-{{\bf{r}}}_{0}|}^{3}},F={\rm{const}},$$

where **r**_0_ is the radius vector of the point defect in a crystal.

The differential Eq. (3) describing the diffracted wave propagation through an imperfect crystal can be cast in the integral form

6 $$\begin{array}{rcl}{E}_{h}({S}_{0},{S}_{h}) & = & {E}_{h}^{(id)}({S}_{0},{S}_{h})\\  &  & +\,{\int }_{-{S}_{0}}^{{S}_{h}}\,d{P}_{h}\,{\int }_{-{P}_{h}}^{{S}_{0}}\,d{P}_{0}{K}_{hh}^{(id)}({S}_{h}-{P}_{h},{S}_{0}-{P}_{0})\\  &  & \times \,\frac{\partial }{\partial {P}_{0}}[\frac{\partial }{\partial {P}_{h}}[if({P}_{0},{P}_{h})]{E}_{h}({P}_{0},{P}_{h})],\end{array}$$

where the *dynamical* diffracted wave amplitude ${E}_{h}^{(id)}({S}_{0},{S}_{h})$ within a perfect crystal and the kernel function ${K}_{hh}^{(id)}({S}_{h}-{P}_{h},{S}_{0}-{P}_{0})$ take the form (see, *e.g*.^1,3^ for details)

7 $$\begin{array}{rcl}{E}_{h}^{(id)}({S}_{0},{S}_{h}) & = & \frac{i{{\Gamma }}_{h}}{{\Gamma }}\,\sin [({S}_{0}+{S}_{h}){\Gamma }],\,({S}_{0}+{S}_{h}\ge 0),\\ {K}_{hh}^{(id)}({S}_{h}-{P}_{h},{S}_{0}-{P}_{0}) & = & {J}_{0}(2{\Gamma }\sqrt{({S}_{h}-{P}_{h})({S}_{0}-{P}_{0})}),\end{array}$$

respectively.

In the Eq. (7), ${E}_{h}^{(id)}({S}_{0},{S}_{h})$ and ${K}_{hh}^{(id)}({S}_{h}-{P}_{h},{S}_{0}-{P}_{0})$ are the corresponding diffracted wave amplitude and the Green (point source) function in a perfect crystal; ${J}_{0}(2{\Gamma }\sqrt{({S}_{h}-{P}_{h})({S}_{0}-{P}_{0})})$ is the zero-order Bessel function of the first kind.

Further, we will use the image peculiarity of the XRDT 2D projections that are directly linked with strongly distorted regions near around a single defect core. As is shown in^1–3^, in such the regions the X-ray diffraction scattering is, in general, kinematical owing to the interbranch scattering of the Bloch X-ray waves. Physically, it does mean that the so-called *direct* defect image contrast on the XRDT 2D projections is due to the diffracted wave propagation through the strongly distorted crystal region along the wave vector **k**_*h*_. It immediately follows that the *direct* defect image on the XRDT 2D projections is formed due to the *kinematical* scattering.

The above assertion is equivalent to building the first-order perturbation theory solution of the *dynamical* Takagi-Taupin Eq. (1) if the corresponding zero-order approximation for the X-ray transmitted amplitude in a perfect crystal is used.

Thus, after some obvious straightforward manipulations with the integral Eq. (6), one obtains the following expression

8 $${E}_{h}({S}_{0},{S}_{h})=i{{\Gamma }}_{h}\,{\int }_{-{S}_{0}}^{{S}_{h}}\,d{P}_{h}\,{e}^{-if({S}_{0},{P}_{h})}{E}_{0}^{(id)}({S}_{0},{P}_{h}),$$

for the diffracted wave amplitude in the scope of the *semi-kinematical* approach, where the *dynamical* transmitted wave amplitude ${E}_{0}^{(id)}({S}_{0},{P}_{h})$ within a perfect crystal is defined by

9 $${E}_{0}^{(id)}({S}_{0},{S}_{h})=\,\cos [({S}_{0}+{S}_{h}){\Gamma }],\,({S}_{0}+{S}_{h}\ge 0).$$

and the integral in the right-hand side of Eq. (8) is taken over the variable *P*_*h*_ along the direction of the diffracted wave propagation.

Formula (8) for the amplitude ${E}_{h}({S}_{0},{S}_{h})$ describes the *kinematical* scattering of the X-ray *dynamical* transmitted wave with the amplitude ${E}_{0}^{(i{\rm{d}})}({S}_{0},{P}_{h})$ ain vicinity of the defect core, which represents by itself the pure phase object. That is why the formula (8) can be treated as the *semi-kinematical* approximate solution of the *dynamical* Takagi-Taupin Eq. (1).

Further, the theoretical formula (8) rewritten as

10 $${E}_{h}({S}_{0},{S}_{h})=\frac{i{{\Gamma }}_{h}}{\cos \,{\theta }_{B}\,\cos \,{\Phi }}\,{\int }_{0}^{T}\,dZ{e}^{-if(X(Z),Y(Z),Z)}{E}_{0}^{(id)}(X(Z),Z),$$

is utilized for recovering the 3D displacement-field function $f({\bf{r}}-{{\bf{r}}}_{0})={\boldsymbol{h}}\cdot {\bf{u}}({\bf{r}}-{{\bf{r}}}_{0})$ together with the corresponding expression for function $f({\bf{r}}-{{\bf{r}}}_{0})$, namely:

11 $$\begin{array}{rcl}f({\bf{r}}-{{\bf{r}}}_{0}) & = & G\tfrac{X+\tfrac{{\rm{tg}}{\theta }_{{\rm{B}}}}{\cos \,\Phi }(Z-{Z}_{0})}{{({(X+\tfrac{{\rm{tg}}{\theta }_{{\rm{B}}}}{\cos \Phi }(z-{z}_{0}))}^{2}+{(Y+{\rm{tg}}{\Phi }(Z-{Z}_{0}))}^{2}+{(Z-{Z}_{0})}^{2})}^{3/2}},\\ G & = & {\rm{const}}.\end{array}$$

where *Ф* is the rotation angle around the diffraction vector ***h*** (see Fig. 1). Note that the dimensionless *X*, *Y*, *Z* coordinates are linked with the intrinsic Cartesian coordinates in the crystal.

The formulas (10), (11) for the 3D displacement-field function $f({\bf{r}}-{{\bf{r}}}_{0})$ are the basic theoretical expressions for solving the inverse problem XRDT under consideration. Finally, one has to determine the error-functional (the target function) and generate its optimization procedure. The latter has to apply to the target function in the standard form as follows

12 $$\begin{array}{rcl}{\rm{\chi }}2 & = & \tfrac{1}{n\{X(T)\,\ast \,Y(T)\}}\,\mathop{\sum }\limits_{i=1}^{n}\,n({{\Phi }}_{i})\,\sum _{\{X(T),Y(T)\}}\,\tfrac{{({I}_{h,obs}[X(T),Y(T);{{\Phi }}_{i}]-{I}_{h,calc}[X(T),Y(T);{{\Phi }}_{i}])}^{2}}{{I}_{h,obs}{[X(T),Y(T);{{\Phi }}_{i}]}^{2}}=Min,\\ n({{\Phi }}_{i}) & = & 1,i=1,\ldots n,\end{array}$$

where the rectangular prism with dimensions 0 ≤ *Z* *≤* *T*, −X/2 ≤ *X(T)* *≤* X/2, −Y/2 ≤ *Y*(*T*) ≤ Y/2, summation in the right-hand side of Eq. (12) is carried out over an array of the XRDT 2D-projections, *n* is the number of the inclined XRDT 2D-projections; the dimensionless thickness T is chosen to be equal to unity.

### Recovery of the 3D displacement-field function. The SA and qNGD algorithm codes

In this Section, we apply the iterative SA^16^ and qNGD^17,18^ algorithm codes, adapted to solving the inverse XRDT problem.

### SA algorithm: setting the displacement-field function in analytical form

As is known, the SA algorithm^16^ is applied to minimize nonlinear target χ^2^-functions. Essentially, it is one of the efficient methods for solving inverse problems with a large number of variables and combinatorial nature of iterative calculations. Starting with a model specified as the initial model and varying pseudo-randomly its parameters, the SA algorithm works until the current model fits best the data set ‘observed’ that means the minimum of the target function is achieved. The advantage of the SA algorithm seems to overcome local minima of the target function, which are the main obstacles for other nonlinear optimization methods, *e.g*., for the qNGD methods^17,18^.

In our case, the position of a point defect is set by a radius vector **r**_0_ = **n***T*/2, where **n** is the internal normal to the input crystal surface, *Z* = 0. Note, the crystal thickness *T* of a single crystal Si(111) is chosen such that the X-ray absorption in a sample can be neglected. In the first study stage, one needs to checkup that the SA algorithm code can be effective in the case of one XRDT 2D projection when only the term with *Φ* = 0 is taken into account in formula (12).

Correspondingly, formula (12) reduces to

13 $$\begin{array}{rcl}{{\rm{\chi }}}^{2} & = & 1/N\{X,Y,Z\}\sum _{\{X(T),Y(T)\}}\,\tfrac{{({I}_{h,obs}[X(T),Y(T);0]-{I}_{h,calc}[X(T),Y(T);0])}^{2}}{{I}_{h,obs}{[X(T),Y(T);0]}^{2}}=\,{\rm{Min}}\,,\\ {I}_{h,calc}\{X(T),Y(T);0\} & = & |{E}_{h}\{X(T),Y(T);0\}{|}^{2},\end{array}$$

where ${I}_{h,true}\{X(T),Y(T);0\}$ is the true X-ray topography 2D projection simulated according formula (10) with the true displacement-field function (11). The intensity ${I}_{h,calc}\{X(T),Y(T);0\}$ is calculated according to formula (10) with trial displacement-field functions according to an iterative procedure of the target function optimization.

For simplicity, without the loss of generality, the scaling coefficient G can be set to unity in formula (11). Besides, to estimate the convergence of the iterative optimization procedure, one uses the error parameter CP, which is defined as

14 $${\rm{CP}}=1/N\{X,Y,Z\}\,\sum _{\{X,Y,Z\}}\,\begin{array}{c}\frac{|{f}_{true}\{X,Y,Z;0\}-{f}_{calc}\{X,Y,Z;0\}|}{|{f}_{true}\{X,Y,Z;0\}|}\end{array}.$$

and it yields the quantitative estimate of the relative deviation of a current solution ${f}_{calc}({\bf{r}}-{{\bf{r}}}_{0})$ from the true solution ${f}_{true}({\bf{r}}-{{\bf{r}}}_{0})$.

**Figure 2:**
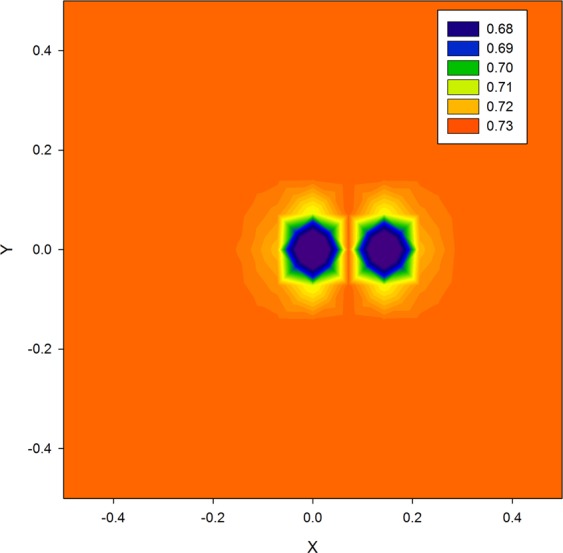
True XRDT 2D imaging projection. The Coulomb-type point defect in a single crystal Si(111). The true displacement-field function with a descending index *p* = 1.5 is determined on the spatial grid nodes {15, 15, 15}, −0.5 ≤ {*X*, *Y*} ≤ 0.5. The rotation angle *Φ* = 0.

**Figure 3:**
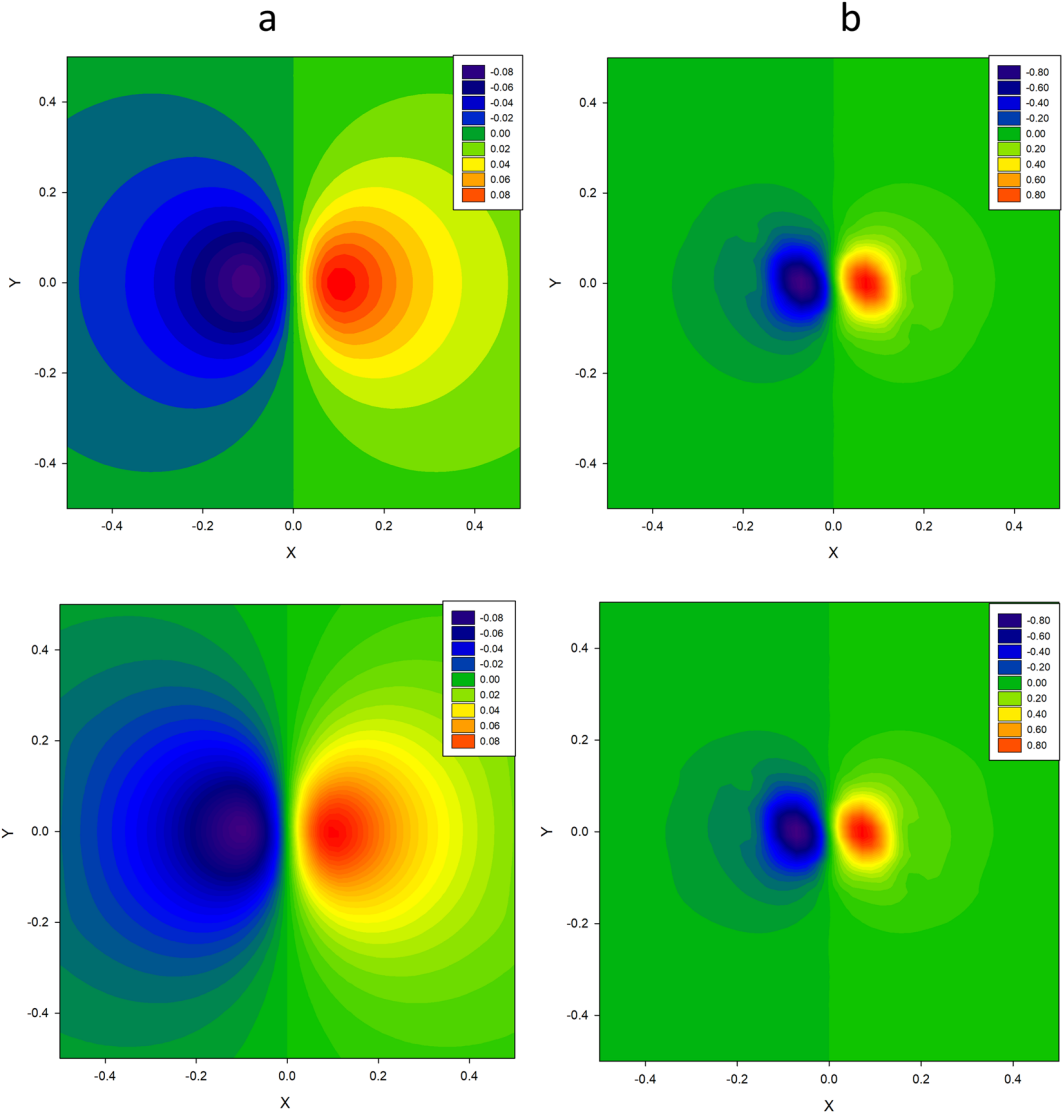
Cross-sections of the 3D displacement-field function in the spatial grid nodes {15, 15, 15}, respectively, −0.5 ≤ {*X*, *Y*} ≤ 0.5, at Z-levels: (**a**) *Z* = 0.375; (**b**) *Z* = 0.5. The upper and lower rows: the cross-sections of the true and recovered 3D displacement-field functions by the qNGD algorithm.

The true XRDT 2D projection is depicted in Fig. 2. As is seen from Fig. 2, the calculated true XRDT 2D projection represents by itself the ‘double form’ image of the Coulomb-type point defect in accordance with the dipole behavior of the function ${f}_{true}({\bf{r}}-{{\bf{r}}}_{0})$ (see Eq. (11), Fig. 3, and^19^ for details).

The initial model of the 3D displacement-filed function ${f}_{ini}({\bf{r}}-{{\bf{r}}}_{0})\,$has been taken as the averaged superposition of functions $f({\bf{r}}-{{\bf{r}}}_{0})$ with the descending indices {*p*_*i*_}, *i* = {1 ÷ 4}. For each initial descending indices combination of *p*_*i*_, *i* = {1 ÷ 4}, the operation range of descending indices *in search* has been chosen in the 3∙Random [*Real*, 0, 1] interval. For particular computer calculations, the spatial grid {41 × 41 × 41} for the Cartesian system coordinates (X, Y, Z) is used.

The recovery data of the SA algorithm code application for the 3D displacement-field function ${f}_{sol}({\bf{r}}-{{\bf{r}}}_{0})$ are listed in Table 1. As it follows from Table 1, in the case of the one descending index *p*_*i*_ for *i* = 1, the solution for the 3D displacement-field function ${f}_{sol}({\bf{r}}-{{\bf{r}}}_{0})$ has been obtained with the accuracy, when the error parameter CP reaches the value of 10^−6^. In the calculated cases of the two with combinations of descending indices *p*_*i*_ for *i* = {1 ÷ 3} and *i* = {1 ÷ 4}, the solution ${f}_{sol}({\bf{r}}-{{\bf{r}}}_{0})$ has been obtained with the error parameter CP of the order of 10^−2^.

**Table 1:** Initial and final recovery data for the 3D displacement-field function by the SA algorithm.

Spatial grid {*i*, *j*, *k*}	Initial			Final		
	*p*_*i*_, {*i* = 1 ÷ 4}	Target Function	CP	*p*_*i*_, {*i* = 1 ÷ 4}	Target Function	CP
{41, 41, 41}	{0.9}	0.79	1	{1.5}	8 · 10^−7^	4 · 10^−7^
{41, 41,41}	{0.5, 1.0, 1.8}	0.48	0.96	{1.49, 1.49, 1.52}	3 · 10^−4^	1 · 10^−2^
{41, 41, 41}	{0.9,1.2,1.8,2.1}	1.44	1	{1.48,1.54,1.49, 1.49}	3 · 10^−3^	1.4 · 10^−2^

The initial 3D displacement-field function parameters {*p*_*i*_}, *i* = {1 ÷ 4}. The 3D displacement-field function is determined in the analytical form.

### SA, qNGD algorithm codes: numerical displacement-field function assignment

From the practical viewpoint, it is of great interest to perform the 3D displacement-field function $f({\bf{r}}-{{\bf{r}}}_{0})$ recovering with its numerical assignment. In contrast to the case above described, we consider the 3D function $f({\bf{r}}-{{\bf{r}}}_{0})$ values in each voxel of the spatial grid as parameters *in search*. Further, one uses the constraints for these functions $f({\bf{r}}-{{\bf{r}}}_{0})$ that are due to the symmetry properties of the function ${f}_{true}({\bf{r}}-{{\bf{r}}}_{0})$ over the variables *X*, *Y*, and *Z* − *Z*_0_. Besides, a requirement for a monotonic decrease of the function ${f}_{true}({\bf{r}}-{{\bf{r}}}_{0})$ with an increase of variables |*X*|, |*Y*|, and |*Z* − *Z*_0_| is imposed as well.

Based on formulas (10), (11), the true XRDT 2D projection for a single crystal Si(111) sample calculated on the spatial grid {15, 15, 15} is displayed in Fig. 2. As is easily seen from Fig. 2, the XRDT 2D projection is symmetric towards the coordinate *Y* and shifted from the center along the *X* coordinate by *T*/2 · tan *θ*_B_. The recovery data of the 3D function ${f}_{sol}({\bf{r}}-{{\bf{r}}}_{0})$ linked with the true XRDT 2D projection are listed in Table 2. The grid node numbers along the 0Z axis, in which the values of the function $f({\bf{r}}-{{\bf{r}}}_{0})$ are altered in the optimization procedure, are shown in bold. Correspondingly, all the other $f({\bf{r}}-{{\bf{r}}}_{0})$ values are chosen as the ones of the true function ${f}_{true}({\bf{r}}-{{\bf{r}}}_{0})$. As it follows from Table 2, in the case of the spatial grid {15, 15, 15} the SA algorithm code application provides the target function value to be reduced by more than six orders of magnitude, whereas the error parameter CP increases from 0.0658 to 0.118. This may occur because of the solution ambiguity of the inverse XRDT problem under consideration.

**Table 2:** Initial and final recovery data of the 3D displacement-field function by the qNGD and SA algorithms.

Spatial grid {*i*, *j*, *k*}	Initial			Final	
	*p*_*ini*_	Target function	CP	Target function	CP
{15, 15, 1–6|**7**–**9**|10–15}	qNGD algorithm				
	1.55	2.52 · 10^−11^	1.77 · 10^−2^	5.67 · 10^−14^	1.81 · 10^−2^
	1.45	1.21 · 10^−11^	1.71 · 10^−2^	6.88 · 10^−21^	4.90 · 10^−6^
	SA algorithm				
	1.55	1.67 · 10^−2^	0.0161	1.31 · 10^−5^	0.0171
{15, 15, 1–5|**6**–**10**|11–15}	qNGD algorithm				
	1.55	2.82 · 10^−11^	2.92 · 10^−2^	2.14 · 10^−22^	2.70 · 10^−3^
	1.45	1.47 · 10^−11^	2.79 · 10^−2^	9.56 · 10^−28^	6.00 · 10^−4^
	SA algorithm				
	1.55	1.56 · 10^−2^	0.0273	2.94 · 10^−6^	0.029
{15, 15, 15}	qNGD algorithm				
	1.55	2.42 · 10^−11^	0.0706	5.03 · 10^−24^	0.119
	1.45	1.24 · 10^−11^	0.0665·	7.99 · 10^−17^	0.107
	SA algorithm				
	1.55	1.42 · 10^−2^	0.0658	2.63 · 10^−8^	0.118

The 3D displacement-field function is determined in the numerical form.

We compare the above3D displacement-field function recovery results with the corresponding ones by using the nonlinear qNGD algorithm based on the Levenberg–Marquardt scheme^20^. Further, one utilizes the open access NL2SNO program code as an implementation of the qNGD algorithm (see^18^ for details). The corresponding optimization results of the target function (13) obtained by the qNGD algorithm are listed in Table 2. It is seen that for the spatial grids, *i.e*.: {15, 15, 1–6|**7**–**9**|10–15} and {15, 15, 1–5|**6**–**10**|11–15}, the final target function values of order of 10^−22^ and the error parameter CP ones of the order of 5 · 10^−5^ are obtained. It should be noted that in the case of the spatial grid {15, 15, 15}, the values of the error parameter CP are much more than the initial error CP ones due to the solution ambiguity of the inverse XRDT problem under consideration.

The recovered 3D displacement-field function ${f}_{sol}({\bf{r}}-{{\bf{r}}}_{0})$ is illustrated in Fig. 3, where the cross-sections of the 3D function ${f}_{sol}({\bf{r}}-{{\bf{r}}}_{0})$ are presented for the two values of Z equal to 0.375, 0.5, respectively.

The upper row (Fig. 3a,b) gives cross-sections of the true 3D function ${f}_{true}({\bf{r}}-{{\bf{r}}}_{0})$, the lower row gives cross-sections of the 3D function ${f}_{sol}({\bf{r}}-{{\bf{r}}}_{0})$ recovered by the qNGD algorithm code. The corresponding 2D cross-section images match each other better for the value of *Z* = 0.5. Generally, this indicates that the 3D function ${f}_{sol}({\bf{r}}-{{\bf{r}}}_{0})$ recovery data are much better in the nearer vicinity, Z = 0.5, of the Coulomb-type point defect core.

## Conclusions

The theoretical *semi-kinematical* approach has been developed that describes the X-ray diffraction propagation in the vicinity of the point defect core in a crystal. To solve the inverse XRDT problem the iterative SA and qNGD algorithm codes have been applied.

In the case of the Coulomb-type point defect the recovery of the 3D displacement-field function $f({\bf{r}}-{{\bf{r}}}_{0})$ has been obtained. With certain limitations on a class of the searched functions $f({\bf{r}}-{{\bf{r}}}_{0})$ specified in both the analytical and numerical forms, the iterative SA and qNGD algorithm codes *in use* work well even for the one XRDT 2D projection.

At this point, it should be noted once more that the present *semi-kinematical* approach embraces the inverse XRDT problem even if alternative algorithm codes have to be applied. On the other hand, a convergence of the optimization procedure for the target function based on the SA and qNGD algorithm codes might be improved by exploiting a number of the true XRDT 2D projections and, besides, some modifications of the SA and qNGD algorithm codes *in use*. The question of whether the elaborated theoretical approach overall is effective or whether it should be given up in favor of other approaches, which should be applied instead, remains a good topic for future work.
